# The association between perchlorate in drinking water and height and weight of children and adolescents in Southwest China: a retrospective cross-sectional study

**DOI:** 10.3389/fpubh.2023.1260612

**Published:** 2023-10-04

**Authors:** Hongyu Guo, Shimiao Zong, Li Yong, Yang Jiang, Ling Qin, Liang Zhou, Qiaoqiao Ren, Xufang Gao, Fayang Kang, Wei Huang, Jianyu Chen, Li Zhang

**Affiliations:** ^1^Sichuan Provincial Center for Disease Control and Prevention, Chengdu, China; ^2^School of Public Health, Chengdu Medical College, Chengdu, China; ^3^Chengdu Center for Disease Control and Prevention, Chengdu, China; ^4^Guangyuan Center for Disease Control and Prevention, Guangyuan, China; ^5^Zigong Center for Disease Control and Prevention, Zigong, China

**Keywords:** drinking water, perchlorate, children, adolescents, height, weight

## Abstract

**Objective:**

To investigate the association between the concentration of perchlorate in drinking water and the height and weight of children and adolescents in Sichuan Province.

**Methods:**

Perchlorate in the drinking water of 24 counties in Sichuan Province from 2021 to 2022 was detected and analyzed, 66 drinking water samples were collected, and the content of perchlorate in drinking water during the wet season and dry season was detected by ultra-high performance liquid chromatography in series. The linear mixed effect model was used to estimate the relationship between perchlorate in drinking water and the height and weight of 144,644 children and adolescents, and 33 pieces of local average wage data were used as confounding factors for quality control.

**Results:**

After controlling the age, gender, and local economic situation, we found that the concentration of perchlorate in drinking water increased by 10 μg/L is associated with a 1.0 cm decrease in height and a 1.6 kg decrease in weight in children and adolescents (*p* < 0.05).

**Conclusion:**

The concentration of perchlorate in drinking water may be negatively correlated with the height and weight of children.

## Background

Perchlorate exists in abundance in the environment; it can be found in components of the environment that include water, soil, and the chemosphere (1). The functional group of perchlorate is ClO_4_^−^, which can bind cations and exhibits high stability (2). The existence of perchlorate in the environment comes from two sources: one is natural, such as natural minerals; and the other is man-made, such as rocket fuel or fertilizer (3). The combination of human and non-human activities leads to the accumulation of perchlorate in the environment and exposure, which is extremely stable in the environment. Due to its solubility and stability, it is easy to migrate to soil, water, and plants. The presence of perchlorate in the environment is a potential health risk for people who eat perchlorate contaminated food and water (4).

Previous research has confirmed the negative influence of perchlorate on human health, particularly its effects on the endocrine and cardiovascular systems; the relationship between perchlorate and allergy diseases has also been investigated (5). Perchlorate is a sodium/iodide symporter (NIS) inhibitor that blocks iodide uptake into the thyroid, thus affecting thyroid function. Thyroid dysfunction can adversely affect somatic growth and development in children (6). Most research on the association between perchlorate and the growth of children has been conducted using indirect inference, by studying the influence of perchlorate on the thyroid hormone through physiological experiments (7). However, given the high exposure dose of children in the development stage, it is urgent to pay attention to the content of perchlorate in various environmental media, comprehensively analyze the exposure dose and health risks, and take timely control and governance measures (8). To date, research using direct population epidemiological evidence has been exceedingly rare. Building on previous studies, we conducted a retrospective cross-sectional study to (1) assess the association between perchlorate in drinking water and the growth of children and adolescents using epidemiological evidence and (2) determine the modifying effects of sociodemographic factors (e.g., gender and age) on the aforementioned association.

Zhang (8) carried out a study on perchlorate levels in drinking water of major basins in China from 2009 to 2020. The results showed that perchlorate pollution in the Yangtze River basin was the highest. Sichuan Basin is located in the upper reaches of the Yangtze River, where 10 main tributaries flow into the Yangtze River. The Sichuan Basin is divided into 183 counties with a population of over 80 million and over 10 million students (9). The advantages of a rich water ecosystem and a large population make Sichuan Basin suitable for such a retrospective cross-sectional study.

## Method

### Data collection

**Figure 1:**
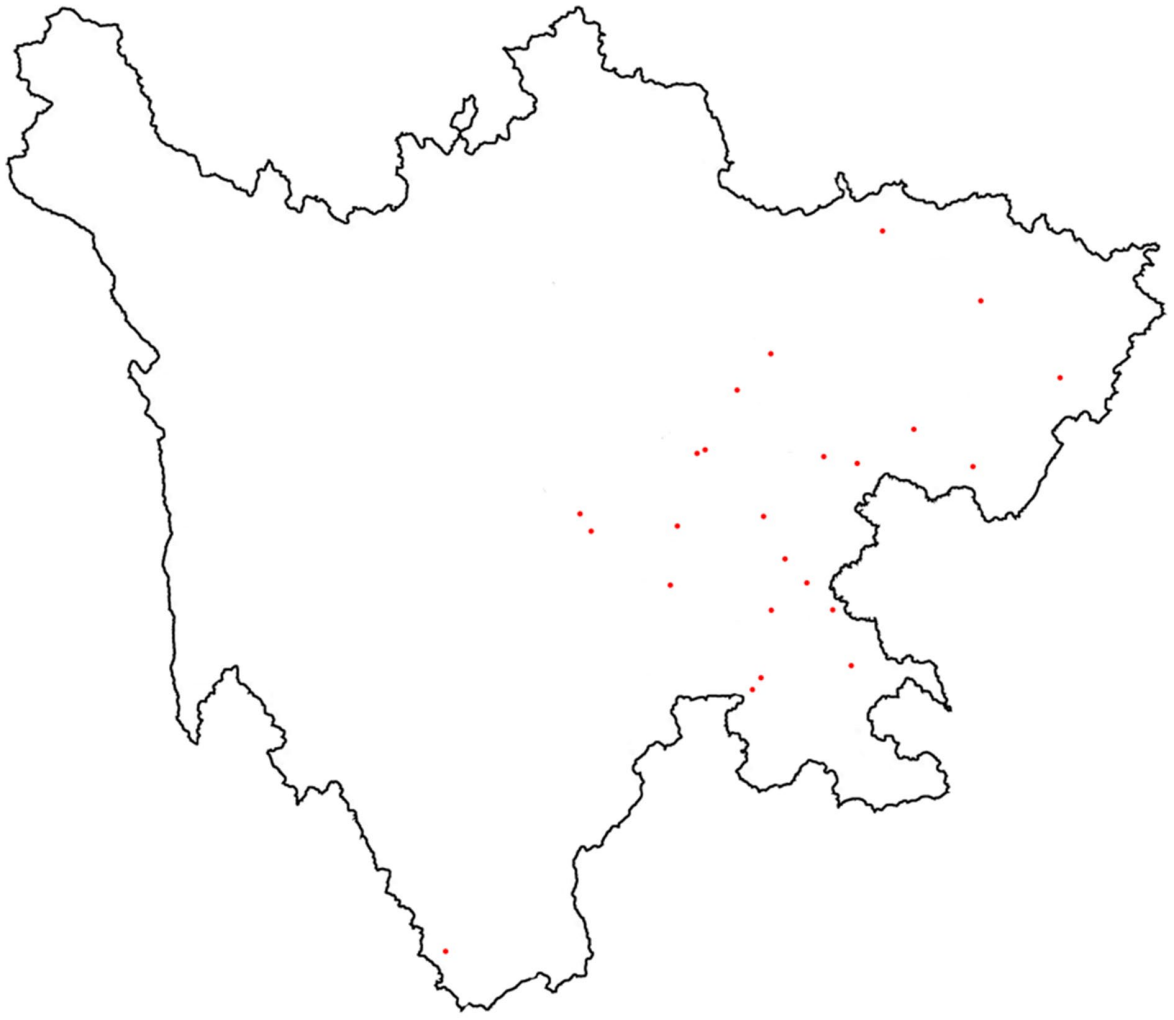
Perchlorate sampling sites in drinking water.

Out of the 183 counties in Sichuan Basin, 24 were selected as study areas during the years 2020 and 2021 (Figure 1). 11 counties were selected for study in 2020 and 24 were selected in 2021; 9 of the same counties were studied in both 2020 and 2021. Two main data points were expected to be obtained in our study: one is the height and weight of children and adolescents as health outcomes; the other is the concentration of perchlorate in drinking water as toxic exposure.

In each county, seven elementary and middle schools were selected each year. From the selected schools, the height and weight of approximately 80 students per grade were monitored once in 2020 and once in 2021. The age of the students in the elementary and middle schools in China ranged between 6 and 18 years. Data from ages below 6 years or above 18 years were omitted in our study. Therefore, the data of height and weight that were obtained correspond to children and adolescents aged between 6 and 18 years of age. Personal information that could identify and locate an individual, such as name, ID number, or phone number, was also omitted in our study. Information regarding gender and age was obtained in this study for subsequent analysis. The primary municipal waterworks that cover the most consumers in Sichuan Basin were selected in each county. During the study, our unified training personnel collected, sealed and transported all perchlorate water samples. To ensure the accuracy of the experimental results, all water samples are tested under the same conditions. We conduct representative selection and analysis twice a year; The first sampling period occurs from July to August during the rainy season, and the second sampling period occurs from November to December during the dry season.

Analyzed for perchlorate in our laboratory using HPLC-MS/MS methods. Perchlorate was analyzed with ACQUITY UPLC I class-XEVO TQ-XS Ultra Performance Liquid Chromatography - Triple Quadrupole Tandem Mass Spectrometer in our laboratory using HPLC-MS/MS methods. In this study, the multi-reaction detection (MRM) mode of ultra-high performance liquid phase spectrometry mass spectrometry was used for detection, and the isotope internal standard method was used for quantitative analysis. The injection amount was 10 μL, and the water sample was filtered by a 0.22 μm aqueous microporous filter membrane, 1 mL of filtrate was injected, and then 5 mL of perchlorate internal standard was added to use the solution, and mixed well for detection. A high concentration range was selected for spiked recovery of perchlorate in drinking water, and 6 replicates were performed at each concentration. The sampling flow rate was controlled at 500 mL/min, about 15 min.

Levels of growth, including the height and weight of children and adolescents, were most likely related to local economic conditions; therefore, data on average local salary each year were utilized in our study as a confounding variable. Data regarding average local salary were collected on the municipal level because there was no such data available at the county level, according to the Statistical yearbook.

All investigations were approved by the Ethics Committee of the Sichuan Provincial Center for Disease Control and Prevention.

### Statistical Analysis

Linear mixed-effects modeling (LME) was applied to calculate the approximate impact of perchlorate on height and weight in our study. Data of height or weight were set as the response variable in the model. The linear predictable variables included both fixed effects and random effects. In our study, the concentration of perchlorate per liter of water was set as the fixed effect predictable variable, while sampling time and county were set as the random effects. The average local salary was set as the confounding variable in the model.

Different subgroups of social demography, including gender groups and age groups, were utilized to assess the effects of sociodemographic factors on the model. The gender groups were stratified into two subgroups, male and female; and the age groups were stratified into 13 subgroups that differ by 1 year in succession (6–18).

To test the statistically significant differences between effect modifiers of sociodemographic factors, a *Z* test was conducted and the equation is as follows:


$$Z=\left(Q_{1}-Q_{2}\right)/\sqrt{{SE}_{1}^{2}+{SE}_{2}^{2}}\text{,}$$

where $Q_{1}$ and $Q_{2}$ are the estimated values of the two categories, while ${SE}_{1}$ and ${SE}_{2}$ are their respective standard errors.

The “NLME” Package in R Program (version 4.2.1) was used to fit our study’s linear mixed-effects model.

## Results

A total of 144,644 children and adolescents were involved in our study, of which 72,174 were male and 72,470 were female. The number of participants for each age group ranging between 6 and 18 years were 9,806, 11,048, 11,498, 10,916, 11,302, 11,144, 11,002, 11,408, 11,428, 13,276, 15,102, 12,392, and 4,322. A total of 66 tap-water samples were gathered and analyzed and 33 average local salaries were utilized in our study. The mean value, standard deviation, and range for height, weight, concentration of perchlorate, and average local salary are listed in Table 1.

**Table 1:** Data for concentrations of perchlorate in drinking water and growth of students aged between 7 and 18 in 24 counties in Sichuan province from 2020 to 2021.

		Mean	SD	Min.	25%	Median	75%	Max.
Height (cm)		149.5	17.2	92.0	135.0	153.0	163.0	197.0
Gender	Male	151.8	19.0	97.0	135.0	155.0	168.5	197.0
	Female	147.1	14.9	92.0	135.0	152.0	158.5	191.0
Age	6	120.1	5.5	92.0	116.0	120.0	123.6	145.0
	7	125.5	5.9	101.0	121.5	125.1	129.0	154.5
	8	130.9	6.3	97.0	126.5	130.8	135.0	158.5
	9	136.3	6.6	101.0	132.0	136.0	140.5	165.0
	10	142.3	7.3	102.0	137.5	142.0	147.0	167.5
	11	148.6	7.5	120.0	143.5	148.5	154.0	179.0
	12	154.6	7.5	120.0	150.0	154.5	159.5	182.0
	13	159.3	7.5	119.0	154.0	159.0	164.0	191.0
	14	162.2	7.9	100.0	157.0	162.0	167.5	196.0
	15	163.9	8.2	110.0	158.0	163.5	170.0	197.0
	16	164.8	8.4	107.0	158.6	164.6	171.0	191.5
	17	165.3	8.7	103.0	159.0	165.0	172.0	193.0
	18	164.4	8.9	129.0	157.5	164.5	171.0	191.0
Weight (kg)		44.2	15.4	12.3	30.8	44.8	54.8	107.1
Gender	Male	46.2	16.9	14.5	31.1	46.2	58.0	107.1
	Female	42.2	13.5	12.3	30.0	43.9	52.0	93.4
Age	6	23.0	4.4	12.3	20.0	22.0	25.0	56.0
	7	25.7	5.2	15.0	22.0	24.5	28.0	55.2
	8	28.9	6.3	16.4	24.5	27.5	32.0	63.8
	9	32.7	7.7	15.9	27.0	31.0	37.0	77.0
	10	37.3	8.8	19.4	30.8	35.5	42.4	78.0
	11	42.3	9.9	21.0	35.0	40.8	48.0	83.0
	12	47.1	10.6	20.8	39.5	45.4	53.0	95.0
	13	51.2	10.4	23.0	44.0	49.4	56.5	100.0
	14	54.0	10.3	25.0	47.0	52.0	59.1	101.0
	15	55.9	10.5	31.0	48.6	54.0	61.0	107.1
	16	57.2	10.5	25.0	50.0	55.2	62.6	105.5
	17	58.1	10.7	23.8	50.2	56.5	64.0	102.0
	18	57.8	10.5	28.0	50.0	56.0	63.6	101.5
Perchlorate (μg/L)		4.27	3.17	0.05	2.20	3.60	5.50	16.50
Local average salary (¥ by 10 k)		6.58	0.57	5.53	6.21	6.50	6.80	8.36

After controlling the variables of age, gender, and local economic conditions, a 10 μg/L increase of concentration of perchlorate in drinking water was found to be associated with a 1.0 cm decrease of height and a 1.6 kg decrease of weight in children and adolescents.

**Figure 2:**
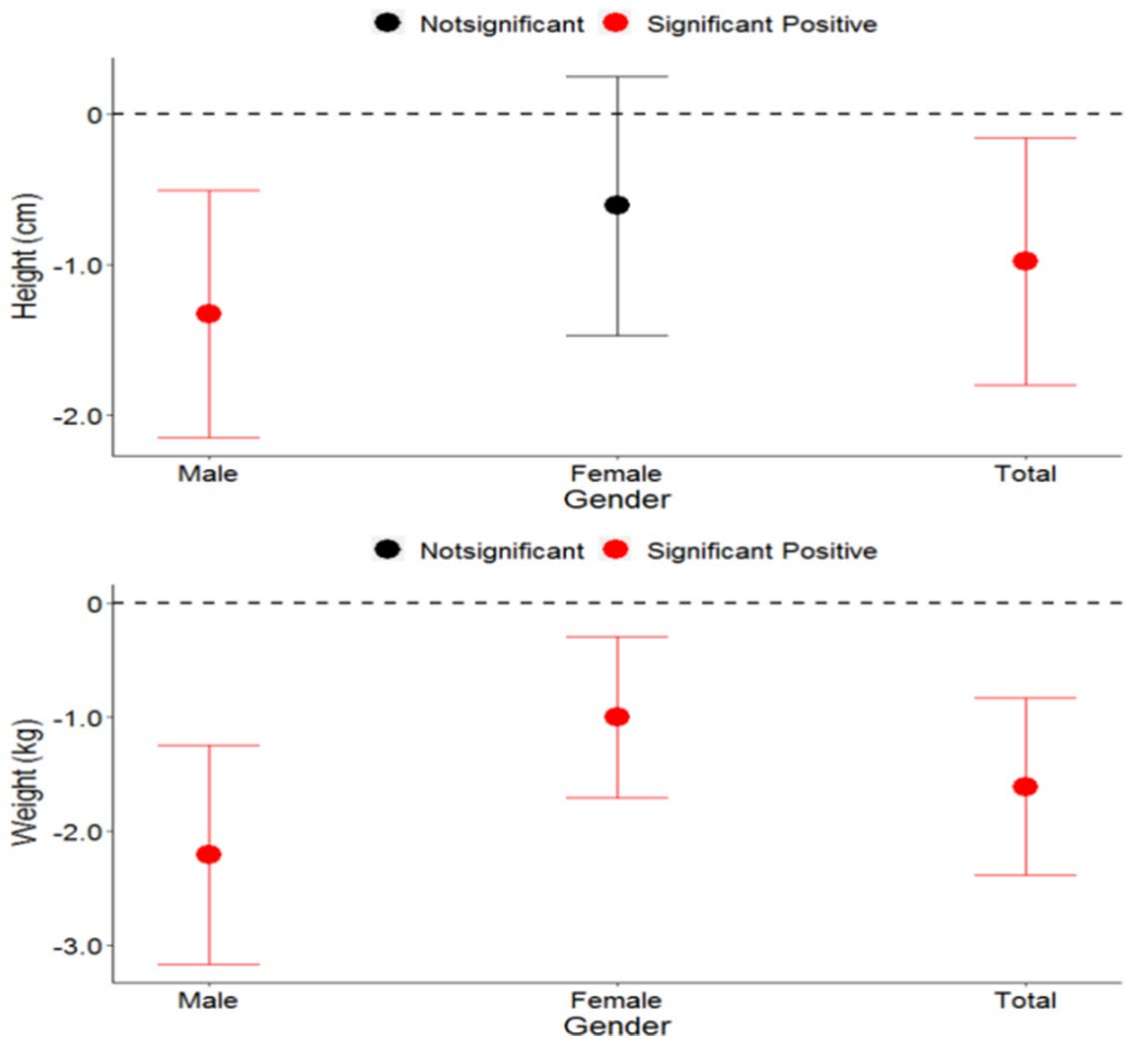
Association between the impact of perchlorate in drinking water on the height and weight and children belonging to various genders.

In males, a 10 μg/L increase of concentration of perchlorate in drinking water was associated with a 1.3 cm decrease of height and a 2.2 kg decrease in weight; in females a 0.6 cm decrease in height and a 1.0 kg decrease in weight. While the decrease in height of males was found to be statistically significant, the decrease in height in females was not of statistical significance. The influence of perchlorate in drinking water on both height and weight was nearly two times higher in males than in females. According to the *Z* test results, the difference in weight decrease between males and females was statistically significant, while the difference in height decrease between the two groups was not (Figure 2).

**Figure 3:**
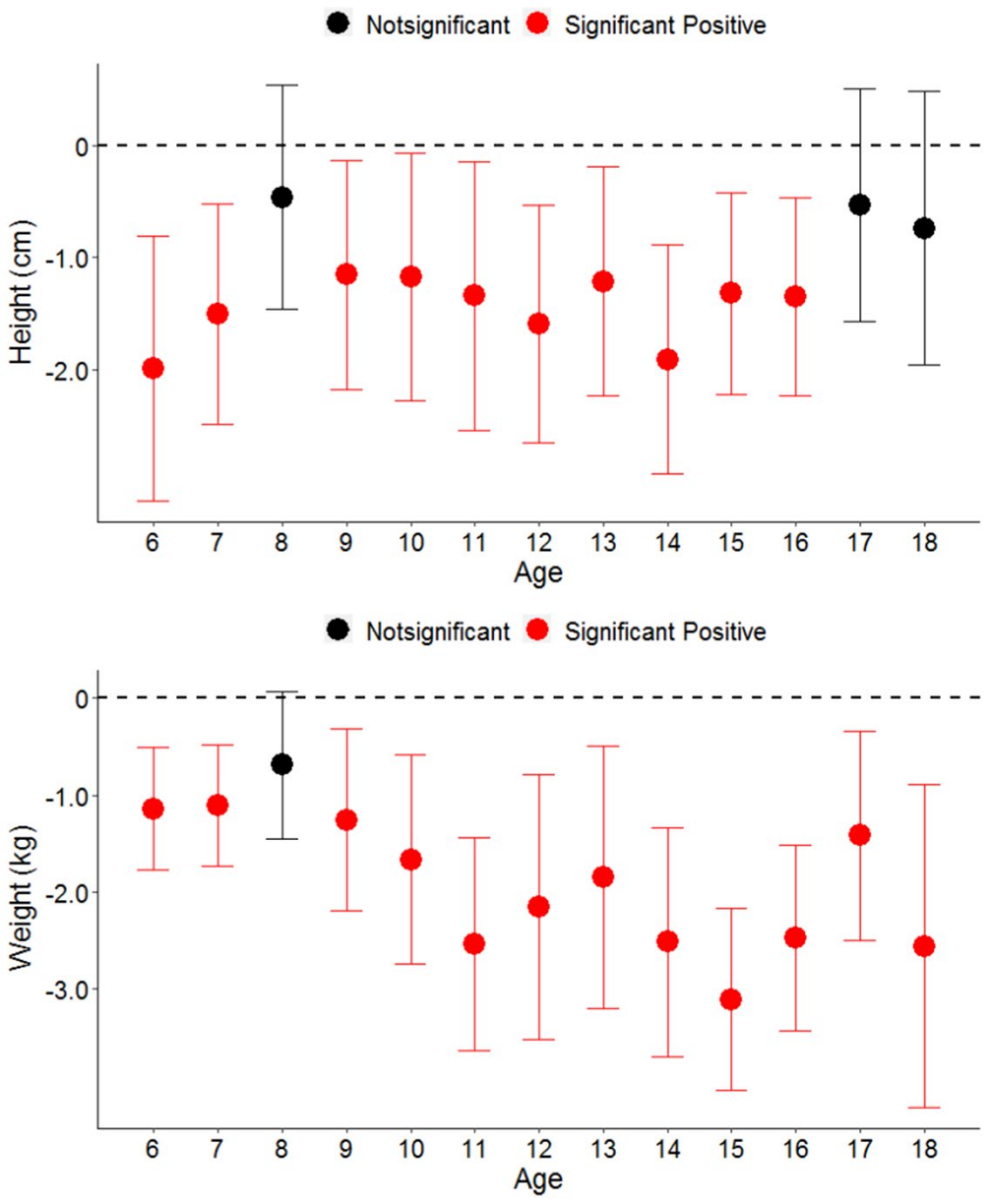
Association between the impact of perchlorate in drinking water on the height and the children across various age groups (6–18 years).

Figure 3 shows the effects of perchlorate on height and weight in different age groups, ranging between 6 and 18 years. The influence of perchlorate in drinking water on height was most obvious in younger children, particularly ages 6 and 7, and adolescents aged ranging between 11 and 14 years. Meanwhile, the influence of perchlorate in drinking water on weight was most obvious in older children, particularly adolescents ranging between 11 and 15 years of age, and those age 18. Changes in height for ages 8, 17, and 18 were not statistically significant, nor were changes in weight for age 8.

Research has found that family economic income is an important factor affecting the growth and development of children and adolescents (10). The average height of children from low-income families is generally lower than that of children from general-income families (11). We included the local urban average wage in the studied areas as a confounding variable, which was associated with changes in both height and weight of children and adolescents according to our study. A 10k CNY increase in average local salary was associated with a 0.6 cm increase in height in children and adolescents, along with a 0.9 kg increase in weight (Supplementary Table S1).

## Discussion

According to the World Health Organization, 80% of diseases worldwide are caused by contaminated drinking water; 50% of the deaths of children around the globe are from contaminated drinking water (12). Research has shown that the health-related quality of life of children and adolescents is affected by height and weight, and children’s height and weight are closely related to environmental pollution, especially drinking water pollution, and its impact is inseparable from human life (13).In this study, we quantified the specific effects of perchlorate in drinking water in children and adolescents at each age stage and found that the height and weight of children and adolescents had a dose–response relationship with perchlorate exposure in drinking water, decreased by the influence of perchlorate in drinking water, and had a statistically significant effect on the weight of adolescents aged 6, 7, and 11–14 years, and adolescents aged 11–15 and 18 years. In the results of this study, no statistically significant effect of perchlorate in drinking water on height and weight at 8, 17, and 18 years of age was found, but there was a downward trend. Studies have shown that because children are sensitive (6, 12, 13), height and weight are more vulnerable than adults to environmental pollutants, and our findings suggest that perchlorate may be harmful to children’s height and weight. Long-term exposure to perchlorate is strongly associated with normal growth rates at different stages of adolescence (14), which may explain the different effects of perchlorate in children and adolescents of different ages in this study. For example, a study in China showed (15) that children (6–11 years) were more affected by perchlorate exposure levels than other age groups (12–79 years). Increased exposure to perchlorate has the greatest impact on children and adolescents (16). Similar and more specific results were obtained, in quantifying the effect of perchlorate on height and weight in children and adolescents, This study found that for every 10 μg/L increase in perchlorate concentration in drinking water, male height decreased by 1.3 cm and weight decreased by 2.2 kg; female height decreased by 0.6 cm in height and 1.0 kg in weight. There was no statistically significant effect on the heights of females. The results of Qin Li ^[18]^also found that perchlorate exposure did not have a significant effect on female height, possibly due to an exposure-response relationship between multiple endocrine-disrupting compounds and male Thyroxine 4(T4) (17, 18). Higher exposure to endocrine-disrupting compounds (perchlorate and other toxins) was associated with lower total T4 levels in men but not in women (19). But according to the results of the National Health and Nutrition Examination Survey (NHANES) (20), perchlorate affects adolescent girls more than the general population. At the same time, This may be related to women’s height and weight in many ways: mental health, genetics, menarche, hormone levels, and different diets between men and women (21–23). Mervish’s study (24) found that girls’ height, waist circumference, and body mass index decreased significantly under high exposure to perchlorate in drinking water (9.6 mg/gC). However, in this study, there may have been no significant effect on female height due to lower perchlorate exposure concentrations (mean: 4.27 μg/L) in drinking water. Most previous studies have focused on the effects of perchlorate in women (3, 19) and no significant effect on height and weight development in adolescent men (7), This study compensated for the lack of data on dose–response relationships in men exposed to perchlorate. Another important conclusion that can be drawn from our findings is that Perchlorate in drinking water affects height and weight twice as much in men as in women. This suggests that perchlorate in drinking water may have a greater effect on growth and development in men than on women and Men’s height and weight development may be more susceptible to low exposure levels of perchlorate in drinking water than women.

As an environmental toxicant, perchlorate has a half-life of 6–8 h in humans (4). Compared with adults, children have immature compound metabolic pathways and smaller blood volumes, which may lead to a prolonged half-life of perchlorate in the body (25). The U.S. Environmental Protection Agency (EPA) added perchlorate to its list of drinking water contaminants in 1998. Because perchlorate interferes with the synthesis, transport, function, and peripheral metabolism of thyroid hormones (26–28), thyroid hormones play an important role in metabolism and control of height and weight development, and long-term obvious thyroxine deficiency can seriously affect children’s height and weight development (29–31). Research has shown a decrease in thyroid hormone levels following exposure to perchlorate (3, 32). For example, for adolescent girls>12, estrogen produced under the influence of perchlorate stimulates changes in serum thyroxine, which can affect women’s thyroid-stimulating hormone (TSH) levels (17). In a study in the United States (25, 33), children with low iodine levels and higher levels of perchlorate exposure had significantly lower thyroid hormones and significantly elevated thyroid-stimulating hormones. Research has shown that (25, 32) that thyroid hormones have a more significant effect on adolescent growth and development than in young children. Therefore, it is reasonable to speculate that perchlorate may indirectly affect height and weight in children and adolescents by affecting thyroid hormone secretion.

Nutritional status in children and adolescents is strongly associated with growth and development (34–36), especially in developing countries, where there is limited diversity in dietary intake among school-age children and adolescents (37), which may affect height and weight development. Studies have shown that households with high household incomes are relatively well nourished, and household income can influence consumption outcomes, which in turn has an indirect effect on children’s height and weight (28). In addition, average local wage as a confounding factor has a significant impact on the height and weight of children and adolescents; this study found that for every 10k CNY increase in local income, children and adolescents experienced an 0.6 cm increase in height and a 0.9 kg increase in weight. This is consistent with the findings of countries such as Japan that children from affluent families have better height and weight development than children from low-income families (26, 38).

Our findings are difficult to compare with previous studies in China, as no previous study has quantified the effect of perchlorate on children’s height and weight. In contrast, our study aimed to quantify the effects of perchlorate exposure on children’s growth and development by analyzing their height and weight. Our findings suggest a stronger association between perchlorate exposure and hazards to children’s growth and development.

Our study has four main strengths. First, the dose–response relationship between perchlorate in drinking water and the growth of children and adolescents located in the upper reaches of the Yangtze River of China was investigated using a linear mixed-effects model. Second, the exposure levels of the subjects’ results were accurate and reliable. Third, the Sichuan Basin is in the upper reaches of the Yangtze River in Southwest China and is characterized by abundant water and a massive population. Safe drinking water is essential to protect public health. Fourth, the present study provides the scientific rationale and epidemic-logic evidence among the population to directly infer the association between perchlorate in drinking water and human growth. It provides valuable data for future studies and efforts to limit the concentration of perchlorate in drinking water. At the same time, the level of perchlorate contamination in drinking water can be monitored and comprehensively evaluated. Several limitations should also be considered: (1) Since this is a cross-sectional study, causality cannot be established, and reverse causation cannot be ruled out; (2) Evaluate single-point water sample collection in the current analysis, and only detect perchlorate concentrations in drinking water during the flood and dry periods, without monthly continuous monitoring, which may be unrepresentative. Therefore, it does not reflect long-term exposure data; (3) According to test data from the US Food and Drug Administration’s Total Diet Study (TDS) (39), perchlorate exposure is not limited to drinking water. Only 21% of food samples contained trace amounts of perchlorate, but it is still important to consider other potential sources such as soil, food, skin, and drug intake. These sources may also cause some interference in our research; (4) Height and weight of adolescents and children are affected by genetics, drugs, and other aspects at the same time, this study only uses the local average wage to represent nutritional status as a confounding factor to control bias. But our research sample size is large and the LME model make up for limitations, therefore, the follow-up should continue to promote the safety supervision of drinking water for children and adolescents, especially in perchlorate and other pollutants should attract enough attention, conditions should be strengthened supervision, monthly investigation and research, further research and analysis are necessary.

In summary, perchlorate in drinking water may have a certain impact on the height and weight of children and adolescents, and it has a certain significance to regulate the safety and healthy development of drinking water for children and adolescents as soon as possible.

## Author contributions

HG: Writing – original draft. SZ: Writing – original draft. LY: Writing – original draft. YJ: Writing – review & editing. LQ: Writing – review & editing. LZ: Writing – review & editing. QR: Writing – review & editing. XG: Writing – review & editing. FK: Writing – review & editing. WH: Writing – review & editing. JC: Writing – original draft. LZ: Writing – review & editing.
