# Supplementary material for: The association between perchlorate in drinking water and height and weight of children and adolescents in Southwest China: a retrospective cross-sectional study
Source: Front Public Health. 2023 Oct 4;11:1260612. doi: 10.3389/fpubh.2023.1260612 (PMC10582749; doi:10.3389/fpubh.2023.1260612)
Supplement: Supplementary file 1 [file Table_1.docx]

Table S1 Linear mixed-effects analysis results of perchlorate on height and weight

| Fixed effect | height 95% (CI) | weight 95% (CI) |
| --- | --- | --- |
| Age |  |  |
| 6 | -2.00(-3.22,-0.83) | -1.13（-1.75,-0.56） |
| 7 | -1.56(-2.56,0.56) | -1.11（-1.69,-0.52） |
| 8 | -0.51(-1.53, 0.48) | -0.69（-1.48, 0.06） |
| 9 | -1.17(-2.11,-0.17) | -1.25（-2.19,-3.52） |
| 10 | -1.22(-2.28,-0.11) | -1.63（-2.73,-0.63） |
| 11 | -1.33(-2.56,-0.17) | -2.51（-3.63,-1.44） |
| 12 | -1.56(-2.67,-0.56) | -2.13（-3.52,-0.81） |
| 13 | -1.22(-2.28,-0.22) | -1.86（-3.16,-0.54） |
| 14 | -1.94(-3.05,-0.89) | -2.44（-3.69,-1.31） |
| 15 | -1.33(-2.21,-0.46) | -3.10（-4.05,-2.15） |
| 16 | -1.39(-2.27,-0.51) | -2.44（-3.38,-1.54） |
| 17 | -0.56(-1,61,0.51) | -1.38（-2.48,-0.38） |
| 18 | -0.77(0.44, 2.05) | -2.54（-4.15,-0.90） |
| Gender |  |  |
| male | -1.32(-2.21,-0.51) | -2.19（-3.09,-1.22） |
| female | -0.57（-1.04, 0.25） | -1.01（-1.64,-0.28） |
| total | -0.99(-1.79,-0.15) | -1.55（-2.32,-0.81） |
| Salary | 0.58(0.09,1.24） | 0.91（0.21,2.64） |
